# Supplementary material for: Imperfect gold standard gene sets yield inaccurate evaluation of causal gene identification methods
Source: Commun Biol. 2024 Jul 17;7:873. doi: 10.1038/s42003-024-06482-1 (PMC11255313; doi:10.1038/s42003-024-06482-1)
Supplement: Supplementary file 2 — Description of Additional Supplementary Files [file 42003_2024_6482_MOESM2_ESM.pdf]

Description of Additional Supplementary Files

File name: Supplementary Data
Description: R-script implementing simulations described in the main text and presented in Figure 3.
